# Concurrent micro-RNA mediated silencing of tick-borne flavivirus replication in tick vector and in the brain of vertebrate host

**DOI:** 10.1038/srep33088

**Published:** 2016-09-13

**Authors:** Konstantin A. Tsetsarkin, Guangping Liu, Heather Kenney, Meghan Hermance, Saravanan Thangamani, Alexander G. Pletnev

**Affiliations:** 1 Laboratory of Infectious Diseases, National Institute of Allergy and Infectious Diseases, National Institutes of Health, Bethesda, Maryland, USA; 2 Department of Pathology, University of Texas Medical Branch (UTMB), Galveston, Texas, USA; 3 Department of Pathology, Galveston National Laboratory, UTMB, Galveston, Texas, USA

## Abstract

Tick-borne viruses include medically important zoonotic pathogens that can cause life-threatening diseases. Unlike mosquito-borne viruses, whose impact can be restrained via mosquito population control programs, for tick-borne viruses only vaccination remains the reliable means of disease prevention. For live vaccine viruses a concern exists, that spillovers from viremic vaccinees could result in introduction of genetically modified viruses into sustainable tick-vertebrate host transmission cycle in nature. To restrict tick-borne flavivirus (Langat virus, LGTV) vector tropism, we inserted target sequences for tick-specific microRNAs (mir-1, mir-275 and mir-279) individually or in combination into several distant regions of LGTV genome. This caused selective attenuation of viral replication in tick-derived cells. LGTV expressing combinations of target sequences for tick- and vertebrate CNS-specific miRNAs were developed. The resulting viruses replicated efficiently and remained stable in simian Vero cells, which do not express these miRNAs, however were severely restricted to replicate in tick-derived cells. In addition, simultaneous dual miRNA targeting led to silencing of virus replication in live *Ixodes ricinus* ticks and abolished virus neurotropism in highly permissive newborn mice. The concurrent restriction of adverse replication events in vertebrate and invertebrate hosts will, therefore, ensure the environmental safety of live tick-borne virus vaccine candidates.

Many of arthropod-borne viruses (arboviruses) are emerging and re-emerging human pathogens that have caused outbreaks of devastating and often fatal diseases and represent a serious public health problem in many regions of the world. During past decades, arboviruses mainly belonging to the *Flaviviridae* and *Togaviridae* families (Chikungunya, Dengue, Japanese encephalitis, tick-borne encephalitis, West Nile and Zika viruses) unexpectedly appeared in new geographic areas of the world where previously they had not been endemic^1^. Compared to the natural transmission cycle of other viruses, arboviruses need to replicate in two hosts: vertebrate and invertebrate vector (mosquito, tick or other arthropod). The spread of these viruses in new areas and the increase in incidence of arbovirus-associated illness highlight the need for efficacious vaccines, antiviral drugs, as well as novel approaches for vector control. Live-attenuated virus vaccines are known to have several favorable advantages compared to other vaccine formulations and induce a long-lasting protective immunity^2^, suggesting that the development of new safe live virus vaccines will help in prevention of arbovirus-caused illnesses, protecting and improving the public health.

Application of live attenuated vaccines (LAV) in endemic and non-endemic areas raises a concern for the environmental safety of the vaccine strains^3^. Vaccine viruses might potentially be introduced into the wild during the feeding of a competent arthropod vector on a viremic vaccinee, as has been shown earlier^4^. Once the virus enters the natural transmission cycle, the adaptive pressures from natural hosts and a high mutation rate of viral RNA-depended RNA-polymerase can inadvertently ‘force’ vaccine virus to rapidly lose the attenuating phenotype, posing a threat to public health and wild life, especially in the areas where virus was not present previously. To address this concern, we showed that targeting of mosquito-borne Dengue type 4 virus (DEN4) genome for mosquito-enriched miRNAs abolishes virus transmission by its natural vectors: *Aedes* (*A*.) *aegypti* and *A. albopictus* mosquitoes^5^.

Suitable for design of environmentally safe vaccine against mosquito-borne viruses, it remains unknown if miRNA targeting approach can restrict vector tropism of tick-borne flaviviruses. This is particularly important since tick-borne viruses exploit not an insect but phylogenetically unrelated arachnid vector for natural transmission. Also it remains unclear whether this approach can be used for development of live vaccine viruses utilizing concurrent restriction of virus replication in the CNS of vertebrate host and invertebrate vector.

There are substantial differences between natural transmission cycles of tick- and mosquito-borne flaviviruses in terms of arthropod vector biology and dynamics of virus infection in ticks and mosquitoes, which potentially can favor transmission of live attenuated (vaccine) strains of tick-borne flaviviruses that cause low, if any, viremia in vertebrate host:

1. The complete life cycle of ticks includes several developmental stages (eggs, larvae, nymphs and adults), and experimental data indicate that after hatching from eggs, ticks might be infected at each stage of their development during feeding on viremic vertebrate host^6^.
2. In contrast to mosquitos, digestion of infectious blood meal in tick midgut occurs intracellularly, limiting the requirement for a high concentration of digestive enzymes in the lumen of the gut^7^. This protects the virus from rapid degradation, providing more time to interact and invade tick midgut epithelial cells. In addition, ticks excrete an excess of water with saliva during feeding, further concentrating virus in the blood meals. As a result, the infection threshold (the virus titer in the blood required for vector infection) for tick-borne viruses is believed to be substantially lower than for mosquito-borne viruses. For example, an infection threshold for tick-borne encephalitis virus (TBEV) was estimated to be 2.0 log_10_ LD_50_/0.02 mL for *Ixodes ricinus* larvae fed on natural host *Apodemus flavicollis*^8^. This corresponds to ∼2–3 log_10_ pfu/mL assuming LD_50_ = 0.1–0.01 pfu^9^,^10^. In contrast, infection threshold of all serotypes of DENV in natural vectors *A.aegypti* and *A. albopictus* was estimated to be 7–8 log_10_ copies/mL^11^, which corresponds to ∼ 4–5 log_10_ pfu/mL.
3. Unlike mosquito-borne viruses, tick-borne flaviviruses can be efficiently transmitted between co-feeding ticks even without the development of a systemic viremia in the vertebrate host [reviewed in ref. 6]. This phenomenon, known as non-viremic transmission, is believed to be mediated by plasma and skin leukocytes of the vertebrate host, and also facilitated by proteins and other compounds introduced with tick saliva into the skin during prolonged feeding on a host^7^ that can last for days or weeks.
4. The transovarial or vertical transmission (TOT) also has been documented for tick-borne flaviviruses^12^,^13^,^14^. Even though occurring at a relatively low rate, TOT nevertheless might additionally contribute to introduction and establishment of genetically modified tick-borne flaviviruses in nature, because vertically infected larvae can substantially proliferate the viral spread in the population of nymphal ticks via non-viremic transmission mode, since larvae are known to quest for hosts synchronously in groups or clasters^15^ [and reviewed in ref. 7].

In the present study, we sought to determine whether the miRNA targeting approach can be used to achieve a selective restriction of vector tropism for viruses that belong to tick-borne flavivirus complex. Naturally attenuated tick-borne Langat virus (LGTV) was selected as a model for highly pathogenic and neurotropic members of the TBEV complex to investigate the effect of miRNA targeting on virus fitness in tick cells and live vector. Isolated in 1950s from ixodid ticks in Malaysia^16^, this virus did not appear to be associated with human diseases and, unlike most members of TBEV complex, requires only biosafety level 2 biocontainment. Moreover, LGTV is being considered as a genetic platform for development of live attenuated vaccines against neurotropic tick-borne flaviviruses^17^,^18^,^19^, underscoring relevance of the virus for vaccine research.

## Results

**Figure 1:**
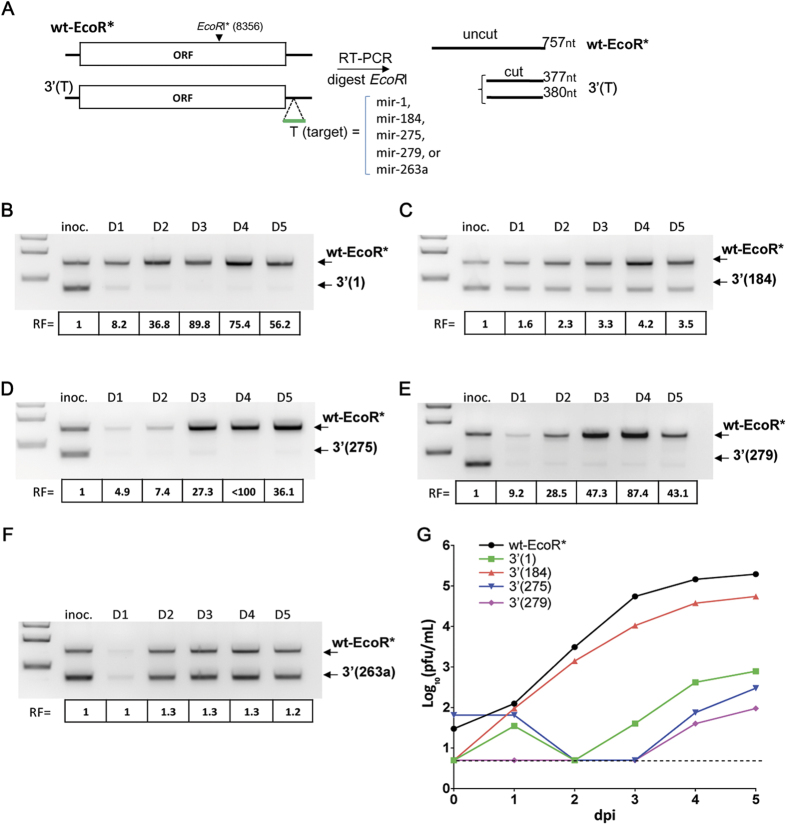
miRNA targeting of the 3′ NCR of LGTV for tick-specific mir-1, mir-275 and mir-279 greatly inhibits virus replication in tick-derived cells. (**A**) Schematic representation of the competition experiment and viral genomes used in the study. ISE6 cells were infected at an MOI of 0.1 pfu/cell with 1∶1 mixture of wt-EcoR* and one of the miRNA targeted LGTV viruses [3′(1), 3′(184), 3′(275), 3′(279) or 3′(263a)]. Supernatants were collected daily until 5 dpi to be used for RNA extraction followed by RT-PCR amplification, EcoR*I* digestion, and gel image analysis. DNA band intensities were quantified to determine the relative fitness (RF) of the competing viruses. RF was determined as the ratio between intensities of DNA bands of amplicons derived from wt-EcoR* and one of miRNA targeted 3′(T) viruses for each time point (D1–D5; dpi) normalized to the starting ratio of viral bands in the inoculum (inoc.) used for ISE6 cells infection. (**B–F**) Effect of insertion of a single target copy for mir-1 (**B**), mir-184 (**C**), mir-275 (**D**), mir-279 (**E**), or mir-263a (**F**) on LGTV fitness in ISE6 cells. (**G)** Growth kinetics of wt-EcoR* and miRNA targeted LGT viruses in ISE6 cells infected at an MOI of 0.01. The dashed line (here and in all subsequent Figures) indicates the limit of virus detection (0.7 log_10_ pfu/mL).

Many miRNAs identified in arthropod species, including mosquitoes and ticks, are evolutionary conservative and abundantly expressed in the different organs during development^20^,^21^ [and reviewed^22^]. Our previous research on the miRNA-mediated control of DEN4 transmission shows that successful knock-down of virus replication in mosquitoes can be achieved by genome targeting only for miRNAs, which are abundantly expressed. To investigate the possibility for selective restriction of LGTV in the ticks, we selected a set of 5 miRNAs (mir-1, -184, -275, -279 and -263a) (Table S1), which have been found to be widely expressed in eggs, larvae and adults (female and male) of cattle ticks *Rhipicephalus microplus*^20^. A sequence complementary to a single copy of each miRNA (target) was inserted into infectious clone of wild-type LGTV strain TP-21 [abbreviated wt^23^;] after the nucleotide (nt) 14 of the 3′ noncoding region (3′ NCR). A single synonymous G→A substitution at position 8356 nt of TP-21 genome was introduced into wt clone, serving as a silent genetic marker. It disrupts the site for *EcoRI* restriction endonuclease in the resulting wt-EcoR* construct, but not in miRNA targeted viruses (Fig. 1A), which allows the differentiation of competing viral genomes. Viruses containing miRNA target for mir-1, mir-184, mir-275, mir-279 or mir-263a in the 3′ NCR and wt-EcoR* were recovered by transfection of plasmid DNA into Vero cells. The kinetics of virus recovery in Vero cells for all miRNA targeted viruses were indistinguishable from wt-EcoR* clone (Fig. S1, p > 0.128 2-way ANOVA, for each virus). In addition, all viruses had similar plaque morphology (~2 mm plaque diameter), indicating that miRNA target insertions do not cause substantial LGTV attenuation in Vero cells, which do not express the above-mentioned miRNAs as determined previously^5^. The region containing miRNA target sequence remained stable in each virus, which was confirmed by sequencing analysis.

To investigate if targeting of LGTV genome for tick-enriched miRNA affects virus fitness in *Ixodes scapularis* derived ISE6 cells, we performed a competition experiment between genetically marked wt-EcoR* and one of each miRNA targeted viruses. For that, each virus with miRNA target sequence in the 3′ NCR was mixed at 1:1 ratio (based on pfu) with wt-EcoR* and resulting concoction was used to infect ISE6 cells at a multiplicity of infection (MOI) of 0.1 pfu per cell. Cell culture supernatants were collected daily followed by virus RNA extraction and RT-PCR analysis with primers flanking *EcoR*I* site (8356 nt). The amplicons were digested with *EcoR*I restriction enzyme and DNA products were analyzed on 1.5% agarose gel. Presence of the mir-1, mir-275, or mir-279 target in LGTV genome resulted in a strong decrease in relative RNA amounts (30–80 folds) by 3 days post infection (dpi) as compared to that of wt-EcoR*. In contrast insertion of targets for mir-184 or mir-263a had a moderate (<5 fold) or negligible (<1.5 fold) effect on LGTV RNA abundance in ISE6 cells, respectively (Fig. 1B–F). This likely reflects the low level expression of these miRNAs in ISE6 cells. To corroborate results of competition experiment, we compared the multicycle replication kinetics of wt-EcoR* and mir-1, -184, -275, and -279 targeted viruses in ISE6 cells infected at an MOI of 0.01. Growth of viruses containing the mir-1, mir-275, or mir-279 target was severely restricted, whereas replication of LGTV with target for mir-184 was only slightly attenuated as compared to wt-EcoR* (Fig. 1G). Based on this data, we selected only target sequences for mir-1, mir-275, and mir-279 for the following studies.

**Figure 2:**
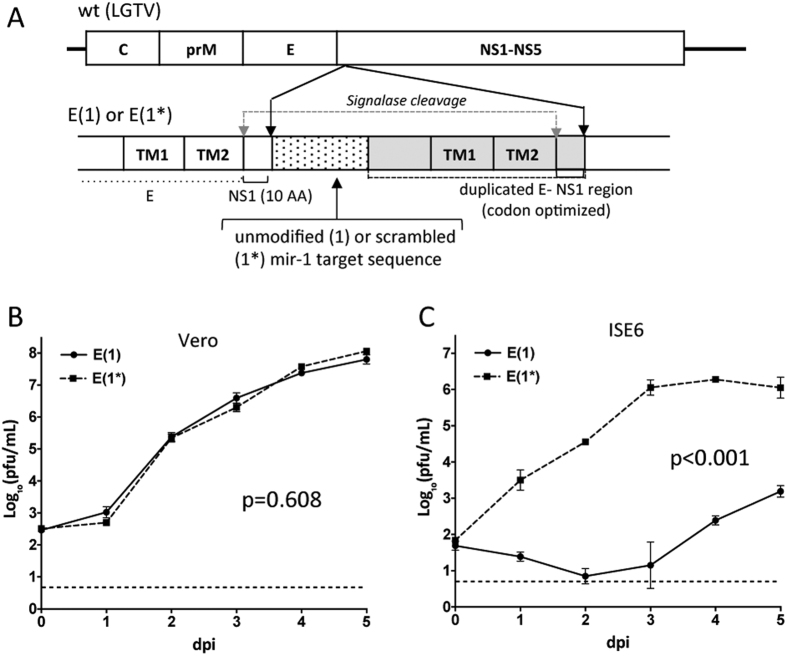
Insertion of a single copy of a target for tick-specific mir-1 into the ORF of LGTV selectively attenuates virus replication in tick-derived ISE6 cells, but not in Vero cells. (**A**) Schematic representation of LGTV miRNA targeted in the dE/NS1R. A mir-1 or scrambled mir-1 (mir-1*) target sequence (pixelated box) was fused with the codon-optimized sequence of an E/NS1 stem-anchor region (2171–2488 nt, gray box) and inserted in-frame at nt position 2489 of the LGTV genome (wt). TM1 and TM2 are transmembrane helixes in the C-terminal stem-anchor region of E protein. (**B**,**C)** Growth kinetics of E(1) and E(1*) viruses in Vero (**B**) and ISE6 (**C**) cells infected at an MOI of 0.01. Titers were determined in Vero cells and presented as an average of two biological replicates (±standard deviation). Differences in growth kinetics were compared using 2-way ANOVA.

To establish if insertion of tick-specific miRNA targets into the open reading frame (ORF) of LGTV genome also results in host-specific attenuation of virus replication, we generated a E(1) virus carrying mir-1 target in the ORF using topology described earlier^24^,^25^. A single target copy for tick-specific mir-1 was introduced in the genome between duplicated sequences encoding the C-terminal stem-anchor domains of E protein and 10 N-terminal amino acid (AA) residues of the NS1 protein (Fig. 2A). A control construct [E(1*)] was generated by introducing synonymous substitutions in each of 7 AA codons that encoded the mir-1 target sequence in the E(1). Replication of E(1) was significantly attenuated as compared to E(1*) in tick-derived ISE6, but not in Vero cells (Fig. 2B,C), indicating that tick-specific miRNA targets remained functional if expressed not only in the 3′ NCR but also in the ORF.

**Figure 3:**
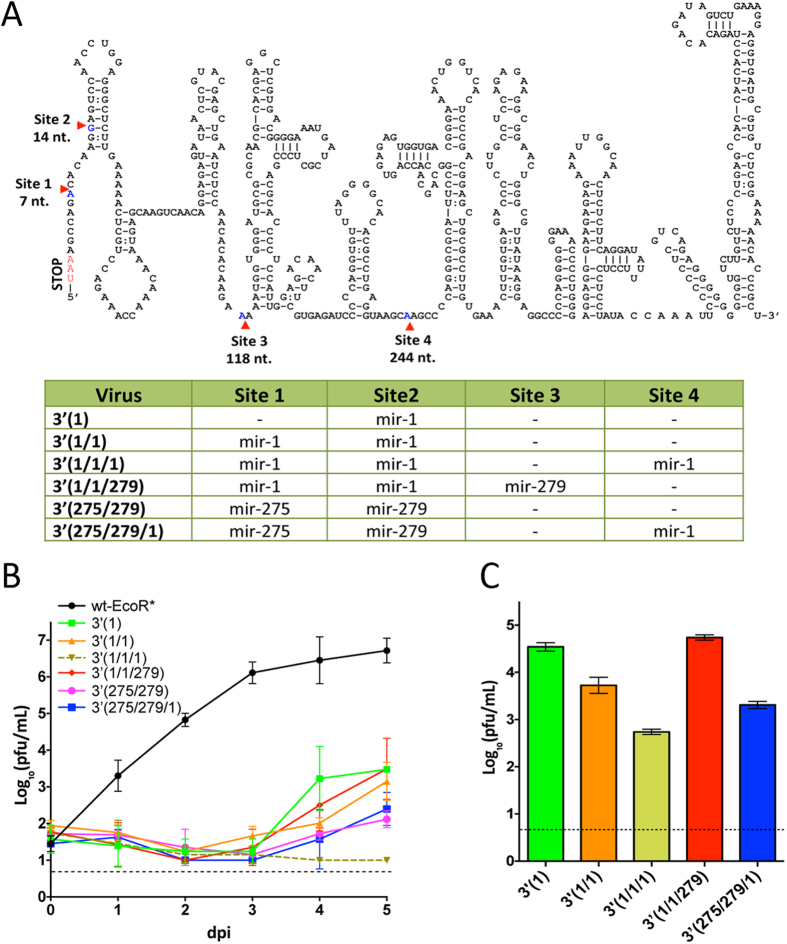
Co-targeting of the 3′ NCR for multiple tick-specific miRNAs does not prevent the emergence of escape mutants in ISE6 cells. (**A**) Schematic representation of recombinant LGTV genomes used in this study. Top: diagram indicating sites for miRNA target insertions in the predicted secondary structure of the 3′ NCR of LGTV. Bottom: recovered viruses with specific composition of the miRNA target(s) inserted into the 3′ NCR. (**B**) Growth kinetics of wt-EcoR* and miRNA targeted LGT viruses in ISE6 cells infected at an MOI of 0.01. (**C**) The titer (±standard deviation) of miRNA targeted viruses after 2^nd^ passage in ISE6 cells. To initiate the 2^nd^ cycle of replication, ISE6 cells were infected at an MOI of 1 and the mean virus titer in the cell supernatants was determined in two replicates in Vero cells.

Insertion of a single target copy for tick-specific miRNA into LGTV was sufficient to greatly inhibit but not completely block virus replication in ISE6 cells, and accumulation of escape mutants lacking miRNA targeted sequences was detected as early as day 3 or 4 p.i. (Fig. 1G). Previously, we showed that insertion of targets for mosquito specific mir-184 and mir-275 into a distant position within the 3′ NCR of DEN4 genome completely blocks virus replication in mosquito-derived Aag2 and C6/36 cells^5^. To explore if insertion of multiple miRNA targets into the 3′ NCR of LGTV can increase virus attenuation in tick cells, we generated a panel of viruses containing two or three copies of homologous (mir-1) or heterologous (mir-1, mir-275, mir-279) targets sequences (Fig. 3A), and compared their replication kinetics in Vero and ISE6 cells infected at a low MOI of 0.01 pfu per cell (Fig. 3B and S2). Only growth of 3′(1/1/279) virus was attenuated in Vero cells compared to wt-EcoR* (Fig. S2, p < 0.001, 2-way ANOVA). In contrast, growth of all miRNA-targeted viruses was greatly attenuated as compared to wt-EcoR* virus in tick ISE6 cells (Fig. 3B, p < 0.001; 2-way ANOVA, for each virus). Moreover, no infectious 3′(1/1/1) virus was detected in ISE6 cell supernatants collected at 4 and 5 dpi. Thus, an increase in the number of mir-1 targets to 2 or 3 copies in the 3′ NCR was associated with gradual decrease of viral growth in the tick-derived cells. To validate this, we infected ISE6 cells with LGTV containing multiple miRNA targets at a high MOI (1 pfu per cell) and performed 2 passages for 5 day incubation each. Regardless of the combination of miRNA targets in the 3′ NCR, all viruses [including the 3′(1/1/1)] grew to the level that was markedly higher than the limit of detection (Fig. 3C). Sequence analysis identified that all recovered viruses lost miRNA targets due to a single or multiple deletions at various locations within the 3′ NCR (Fig. S3), indicating that the selected targeting of the 3′ NCR alone is not sufficient for reliable host range restriction of LGTV during the long-term incubation.

### Generation of LGTV carrying multiple targets for tick- and CNS-specific miRNAs in various genome regions

**Figure 4:**
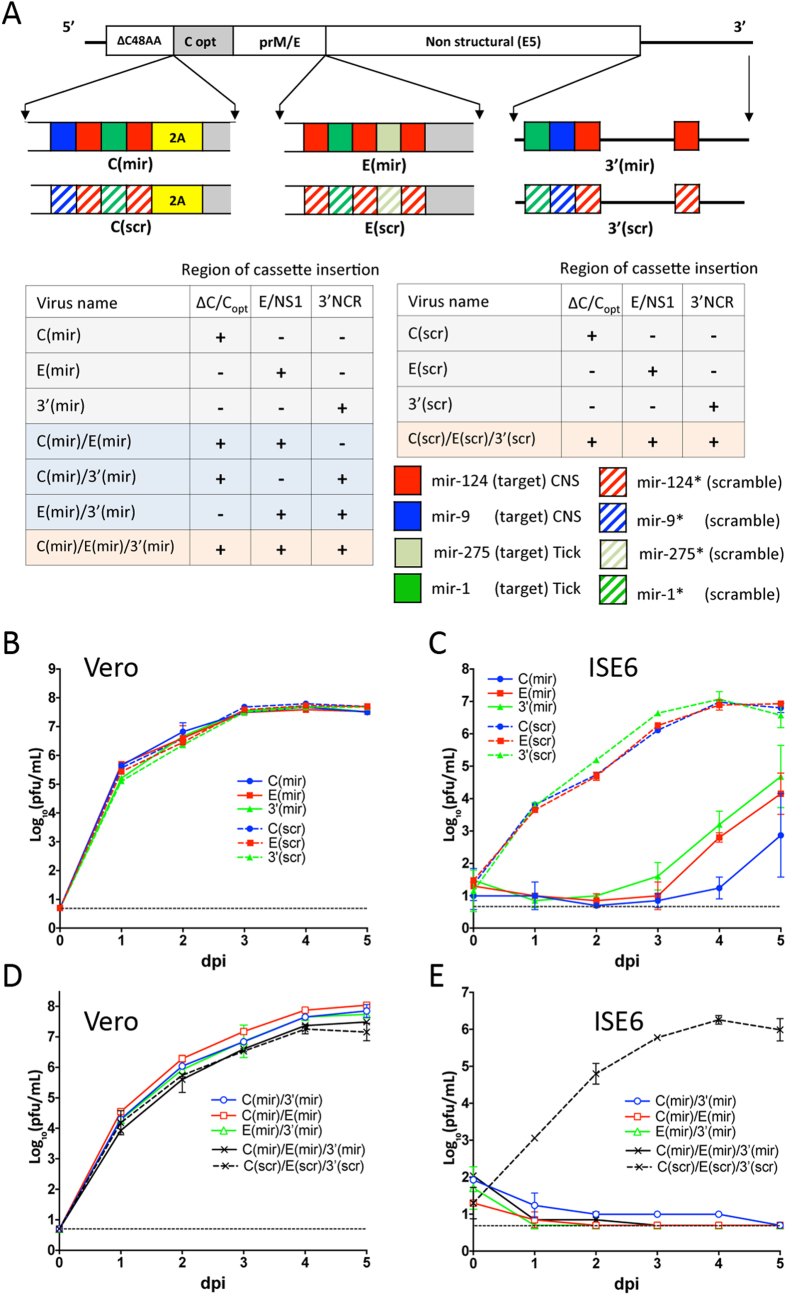
Effect of multiple miRNA targets inserted in distant regions of LGTV genome on virus fitness in ISE6 and Vero cells. **(A)** Schematic representation of viruses used in the study. The miRNA targeting cassettes, which encode targets for tick- and CNS-specific miRNAs (solid boxes), were inserted into the dCGR, dE/NS1R, and 3′ NCR individually or in combinations as indicated (see table at left bottom). For the dCGR insert: miRNA targets were introduced between C48AA region encoding 48 amino acids of truncated C protein and sequence of 2A protease from FMDV (yellow box). Grey areas (**C** opt) represent codon-optimized C protein gene sequence. For E/NS1 insert: a single copy of mir-1 target in E(1) virus (Fig. 2A) was replaced with a set of miRNA-targets as indicated and dE/NS1R was inserted into E5 virus. For the 3′ NCR insert: the indicated miRNA targets were introduced into sites 1–4 as shown in Fig. 3A. Four control (scr) viruses contained multiple substitutions in all miRNA target sequences (striped boxes) within every inserted cassette. (**B**,**D**) Kinetics of infectious virus recovery after plasmid DNA transfection into Vero cells. Plasmids encoding LGTV containing single (**B**) or multiple (**D**) miRNA targeting cassettes and control clones were transfected into Vero cells. Cells culture aliquots were collected daily and titrated in Vero cells in duplicate. (**C**,**D)** Growth kinetics of LGT viruses containing single (**C**) or multiple (**E**) miRNA target cassettes and control (scr) viruses in ISE6 cells infected at an MOI of 0.01.

It was shown that simultaneous miRNA targeting of multiple distant regions within flavivirus genome (ORF and 3′ NCR) increases efficacy of miRNA-mediated virus attenuation as compared to target insertion into a single site^25^. Accordingly, here we sought to determine if insertion of tick-specific miRNA target sequences into multiple locations within LGTV genome can inhibit formation of escape mutants under miRNA-mediated selective pressure. A genome of attenuated LGTV strain E5 (designated E5) was chosen for multiple sites of miRNA-targeting. Strain E5 differs from wt TP-21 strain by five attenuating AA substitutions (N_24_ → S, F_250_ → Y, and F_319_ → L in NS3; T_422_ → S and R_544_ → K in NS5) located in the non-structural NS3 and NS5 proteins^26^,^27^, which could bolster potential application of the resulting viruses as vaccine candidates against encephalitic flaviviruses. Three genome locations were selected for miRNA target insertions (Fig. 4A): duplicated capsid region (dCGR)^23^, duplicated E/NS1 region (dE/NS1R), and 3′ NCR.

To assure concurrent restriction of LGTV neurotropism in vertebrate host and replication in its tick vectors, targets for tick-specific mir-1 and/or mir-275 were inserted in tandem with sequences complementary to CNS-specific mir-124 and/or mir-9 into either dCGR, dE/NS1R, or 3′ NCR and three miRNA-targeted C(mir), E(mir) and 3′(mir) viruses were generated, respectively (Fig. 4A). Control (scrambled) constructs for each of miRNA-targeted viruses contained synonymous substitutions in all of miRNA target sequences at every codon. Target sequences located at the 3′ NCR were mutated at the same nts as target sequences located in the ORF. All miRNA-targeted viruses and scrambled controls replicated efficiently in Vero cells (Fig. 4B), attaining titers of 7.5 log_10_(pfu/mL) at 4–5 dpi and were genetically stable after 10 blind virus passages in Vero cells as was verified by the sequence analysis of viral genomes. To confirm that tick-specific miRNA target(s) remained functional when expressed in a combination with CNS-specific miRNA targets, growth kinetics of C(mir), E(mir) and 3′(mir) were compared to corresponding scrambled control viruses in ISE6 cells infected at an MOI of 0.01. Replication of viruses containing intact miRNA targets was significantly attenuated as compared to scrambled controls (Fig. 4C, p < 0.0001; 2-way ANOVA). To verify these results, we constructed viruses carrying of three target copies for homologous mir-1, mir-9, or mir-124 miRNA (Fig. S4A) and found that replication of LGTV containing three targets for CNS-specific mir-124 or mir-9 in the 3′ NCR was indistinguishable (p > 0.5; 2-way ANOVA) as compared to replication of LGTV control virus with three copies of random sequences in ISE6 cells (Fig. S4A,B). However, replication of LGTV containing three targets for tick-specific mir-1 in the same sites of the 3′ NCR was suppressed in these cells. This demonstrates that presence of intact target sequences for mir-124 and mir-9 in C(mir), E(mir) and 3′(mir) viruses likely resulted only in a negligible effect on viruses attenuation in ISE6 cells, suggesting that LGTV targeting for CNS-specific miRNAs alone does not result in invertebrate-specific host range restriction.

To analyze if co-targeting of distant regions of LGTV genome reduces the risk of escape mutant formation during viral replication in tick-derived cells, we generated viruses by combining miRNA target cassettes from C(mir), E(mir) and 3′(mir) viruses into a single LGTV genome. The resulting viruses contained miRNA targets in all three or in two out of three locations within genome of E5 virus. A control virus C(scr)/E(scr)/3′(scr) contained synonymous substitutions in every miRNA target sequence in all three genome locations (Fig. 4A). All viruses containing multiple miRNA targeting cassettes replicated efficiently in Vero cells (Fig. 4D) and remained stable for at least 10 blind passages in Vero cells. In contrast, accumulation of infectious viruses was not detected in supernatant of ISE6 cells infected with each of miRNA targeted viruses (Fig. 4E). Scrambled control viruses grew efficiently in ISE6 cells indicating that growth restriction of any virus with authentic miRNA-targets in two or three genomic locations was due to a presence of functional miRNA target sequences. Moreover, no infectious viruses were recovered after two blind passages in ISE6 cells that were infected at an MOI of 1 (Fig. S5). In contrast, each virus that contained miRNA target cassettes inserted into only one of the three genome locations was recovered with titers ranging from 2.9 to 4.7 log_10_(pfu/mL). Sequence analysis revealed that replication of these viruses in tick-derived cells was associated with accumulation of deletion mutations that always involved the tick-specific but not necessarily CNS-specific miRNA target sequences (Fig. S5).

### Combined targeting for tick-specific miRNAs in distant genome regions restricts the ability of LGTV to infect *Ixodes ricinus* ticks

**Figure 5:**
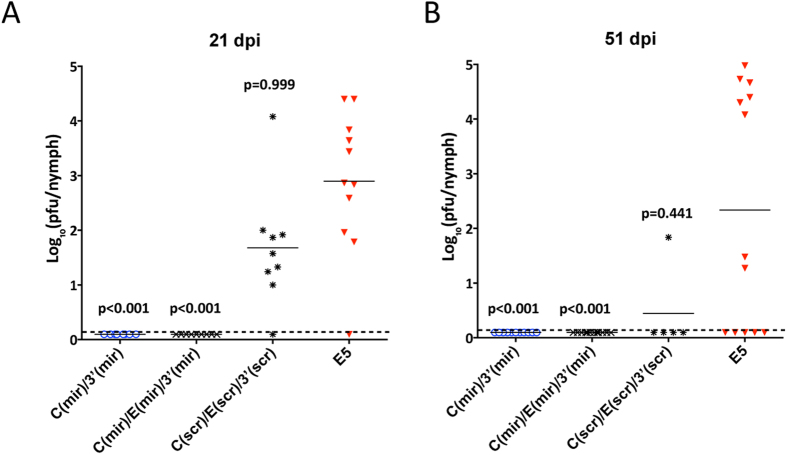
Targeting of LGTV genome for tick-specific miRNAs blocks virus infectivity and replication in *Ixodes ricinus* ticks. *I. ricinus* nymphs were submerged for 45 min in Opti-Pro medium containing ~7 log_10_ pfu/mL of indicated virus and then were returned to glass vials for 21 (**A**) or 51 (**B**) days of incubation. Virus titer in each tick body suspension was determined in Vero cells. Horizontal line represents the mean virus titer for all nymphs in the group. The dashed line indicates the limit of virus detection [0.1 log_10_ pfu/mL]. Differences in infection rates between unmodified E5 virus and one of miRNA targeted [C(mir)/3′(mir) or C(mir)/E(mir)3′(mir)] or scrambled control [C(scr)/E(scr)3′(scr)] viruses were compared using one-tailed Fisher’s exact test, followed by p-values adjustment using Bonferroni correction method to account for multiple comparisons.

To validate results of selective miRNA-mediated LGTV suppression in cell culture, we infected *I. ricinus* nymphal ticks with miRNA targeted viruses C(mir)/3′(mir) or C(mir)/E(mir)/3′(mir) using the synchronous infection method. Control ticks were exposed to C(scr)/E(scr)/3′(scr) or E5 virus that do not have functional miRNA targets (Fig. 4A). Viral titers in tick body homogenates were assayed on 21 and 51 dpi in Vero cells (Fig. 5). Infection rate of parental E5 virus was 91% (n = 11) and 62% (n = 13) on 21 and 51 dpi, respectively, which was not significantly different (p = 0.999 and p = 0.441, one-tailed Fisher’s exact test) from infection rate of another control C(scr)/E(scr)/3′(scr) virus [89% (n = 9) and 20% (n = 5) at 21 and 51 dpi, respectively]. In contrast, none of the ticks exposed to viruses containing intact targets for tick-specific mir-1 and mir-275 became infected (Fig. 5, p < 0.001 for all viruses, one-tailed Fisher’s exact test). Interestingly, the targeting of LGTV genome for only two copies of mir-1 in C(mir)/3′(mir) was sufficient to completely restrict virus vector tropism. It should be noted that although insertion of multiple heterologous sequences into LGTV [C(scr)/E(scr)/3′(scr) vs E5 virus] has the minimal effect on viral infectivity in ticks, however, it caused significant reduction in the viral titer in the infected ticks (Fig. 5A, p < 0.0326, one-tailed unpaired t-test for 21 dpi).

### Co-targeting of viral genome with sequences complementary to tick- and CNS-specific miRNAs does not interfere with miRNA-mediated attenuation of LGTV in the brains of newborn mice

**Figure 6:**
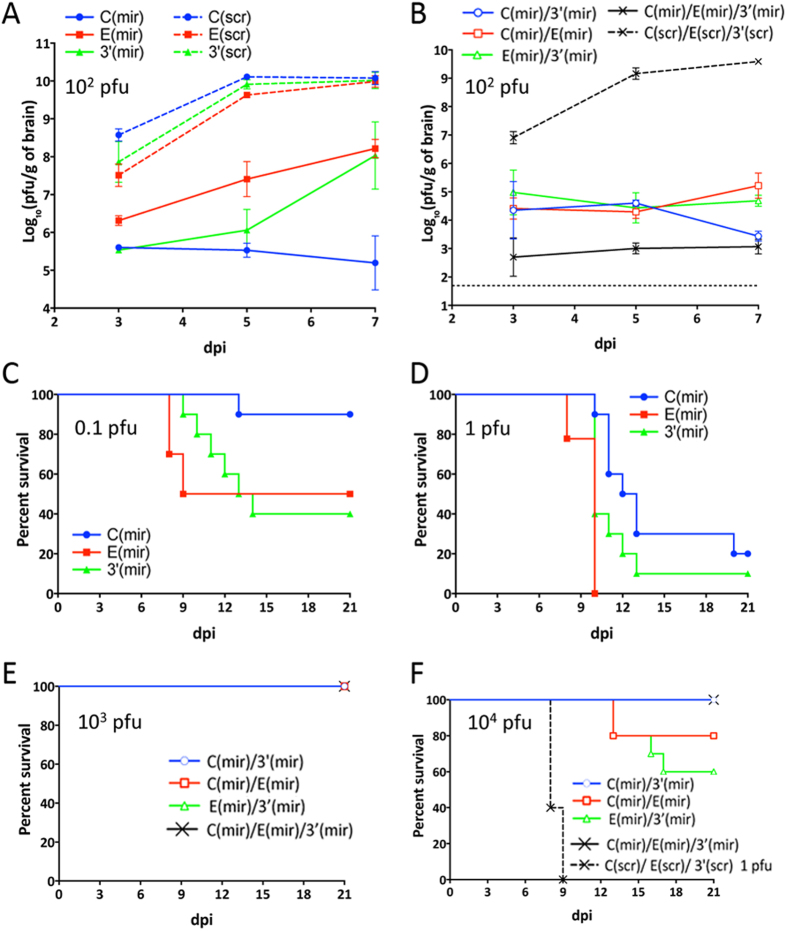
Effect of miRNA targeting cassettes inserted at a single or multiple regions of the LGTV genome on viral pathogenesis and growth in the brain of newborn mice following IC inoculation. (**A**,**B**) 3-day-old Swiss Webster mice were inoculated IC with 10^2^ of LGT viruses containing a single (**A**) or multiple (**B**) miRNA targeting cassettes or containing scrambled control sequences. Mean virus titers (±SD) in the brain of three mice collected at the 3, 5, and 7 dpi are shown. (**C**,**D**) Mice in groups of 10 were inoculated IC with 0.1 (**C**) or 1 (**D**) pfu of C(mir), E(mir) or 3′(mir) virus and monitored daily for morbidity for 21 days. (**E**,**F**) Mice in groups of 10 were inoculated IC with 10^3^ (**E**) or 10^4^ (**F**) pfu of LGT viruses containing multiple miRNA targeting cassettes and monitored daily for morbidity for 21 days. Note: scrambled control virus C(scr)/E(scr)3′(scr) in figure (**F**) was injected at the dose of 1 pfu/mouse.

There is a possibility that insertion of targets for tick-specific miRNAs (mir-1 and mir-275) in combination with targets for CNS-specific miRNAs (mir-124 and mir-9) might prevent microRNA-induced silencing complex (RISC)-dependent attenuation of LGTV replication in the CNS of vertebrates. To explore this probability, we inoculated 3-day-old Swiss mice intracranially with 100 pfu (corresponding to 10^4^ lethal dose 50% for wt LGTV) of viruses containing either authentic [C(mir), E(mir) or 3′(mir)] or mutated [C(scr), E(scr) or 3′(scr)] miRNA targets. Replication of C(mir), E(mir) and 3′(mir) viruses was significantly attenuated in the mouse brain compared to growth rates of scrambled control viruses (Fig. 6A, p < 0.0001; 2-way ANOVA). Interestingly, growth of viruses C(mir) and 3′(mir) in the brains was significantly reduced as compared to E(mir) virus [p < 0.0011 for both pairs; 2-way ANOVA]. This likely indicates that combined expression of targets for two different miRNAs (mir-124 and mir-9) in C(mir) and 3′(mir) results in a stronger virus attenuation in the CNS as compared to the targeting for only mir-124 inserted in the E(mir) genome. Insertion of miRNA targeting cassettes into two or three distantly located regions within LGTV genome led to the additive effect on virus attenuation in the mouse brain and resulted in a 3.3 × 10^6^-fold reduction in the titer of C(mir)/E(mir)/3′(mir) as compared to C(scr)/E(scr)/3′(scr) on 7 dpi (Fig. 6B). To confirm that attenuation of C(mir), E(mir) and 3′(mir) viruses was due to the presence of targets for vertebrate brain-specific mir-124 and/or mir-9 and not due to the presence of targets for tick-specific mir-1 (see Fig. 4A), we compared growth rates in the mouse brain for 3′(1/1/1), 3′(9/9/9) and 3′(124/124/124) viruses carrying of three target copies for homologous mir-1, mir-9, or mir-124 miRNA (see Fig. S4A). The growth rate of 3′(1/1/1) virus with targets for tick-specific mir-1 was indistinguishable from that of a control 3′(gf/gf/gf) virus, which contains three copies of 20 nt sequences from green fluorescent protein (GFP) gene inserted at positions nt. 7, 14, and 244 of the 3′ NCR. However, growth of 3′(1/1/1) virus was significantly higher (p < 0.0001; 2-way ANOVA) as compared to that of 3′(124/124/124) and 3′(9/9/9), which carry target copies for CNS-specific mir-124 or mir-9 in the 3′ NCR (Fig. S4C). This demonstrates that the observed attenuation of viruses in newborn mice was associated with the presence of complementary targets for brain-specific miRNAs in the viral genome. Taken together our data shows that co-targeting of LGTV genome for different miRNAs specifically expressed in the vertebrates and ticks does not affect their function and can result in independent attenuation of virus replication in the ticks and mice brain.

Interestingly, despite the great reduction in virus titer in mouse brains ranging between 10^2^- and 10^4^-fold (Fig. 6A), insertion of a single miRNA targeting cassette in any of the three regions of LGTV genome was not sufficient to prevent mouse morbidity and mortality after IC infection with as little as 0.1 pfu (Fig. 6C,D). However, combined insertion of miRNA targeting cassettes into at least two distant locations within LGTV genome completely prevented morbidity and mortality in highly permissive newborn mice inoculated in the brain with a large dose (10^3^ pfu) of any multiple miRNA co-targeted viruses (Fig. 6E). Moreover, no morbidity or death was recorded in mice infected with a 10^4^ pfu dose of C(mir)/3′(mir) or C(mir)/E(mir)/3′(mir) virus. However, infection with the same dose of C(mir)/E (mir) or E(mir)/3′(mir) virus (both have only mir-124 targets in the dE/NS1R) resulted in 20% and 40% mortality, respectively (Fig. 6F).

To evaluate genetic stability of viruses with multiple miRNA targeting cassettes in the developing CNS, mice were infected IC with the dose 10^4^ pfu and at 7, 13 and 21 dpi brains from 3 or 5 pups were taken for virus isolation and RT-PCR analysis. The infectious C(mir)/3′(mir) and C(mir)/E(mir)/3′(mir) viruses were detected in the brains of mice at 7 dpi but not at 13 and 21 dpi (Fig. S6). Sequence analysis showed that viruses isolated at 7 dpi remained stable as mutations or deletions were not detected at any of the miRNA targeting regions (Fig. S6). In contrast, point mutations and/or single or multiple deletions in the miRNA targeting regions were detected in C(mir)/E (mir) and E(mir)/3′(mir) viruses isolated at each of the time points (Fig. S7). For both viruses the sequence containing a single target for mir-9 was the most unstable, regardless of its position in the virus genome. Based on these observations, we concluded that for the most effective attenuation of LGTV replication in the CNS and to ensure the highest stability of viral genome, targets for two different brain-expressed miRNAs (mir-9 and mir-124) should be expressed simultaneously at two (or more) genome regions.

## Discussion

In this work we demonstrated that miRNA targeting approach aimed at the vector range restriction of arthropod-borne viruses is not to be confined to mosquito-borne flaviviruses^5^, but can also prevent the arthropod transmission of the tick-borne flaviviruses. Targeting of LGTV genome for tick-enriched miRNAs selectively attenuates virus replication in tick-derived cells and blocks virus infection of ixodid tick nymphs. This study serves as a proof of principle that the developed approach can potentially be expanded for silencing replication of other tick-borne viruses, mitigating the risks associated with potential accidental spillovers of genetically modified tick-borne viruses into nature during vaccine campaigns and ensuring the environmental safety of live attenuated virus vaccine candidates.

Findings regarding the miRNA-mediated silencing of LGTV replication in the tick cell line are in an agreement with our data on miRNA-mediated suppression of mosquito-borne DEN4 virus demonstrating that DEN4 genome targeting for mosquito-enriched miRNAs attenuates virus replication in several mosquito-derived cell lines^5^. Thus, both studies indicate that: (1) targets for miRNAs with high arthropod vector abundance [mir-1 in ticks^20^ and mir-275 or mir-184 in mosquitoes^21^] should be used for the effective virus suppression in the respective vector host; (2) target insertions for arthropod vector-specific miRNAs into several distant genome regions are more effective to control virus replication in invertebrate cells than targeting of only single site such as the 3′ NCR (Fig. 4); (3) co-targeting of virus genome for invertebrate vector-specific and brain tissue-enriched miRNA is associated with independent, simultaneous silencing of virus replication in both biological species/tissue types without the interference between miRNA targets (Fig. 4). The latter ensures that this approach can be used to reinforce the safety of LAV against medically important tick-borne viruses.

However, a substantial difference between mosquito/DEN4 and tick/LGTV systems was also observed. For the tick/LGTV system the tick-specific miRNA targets remain equally effective if placed in the 3′ NCR or ORF (Figs 2, 3 and 4). In contrast, for mosquito/DEN4 system, targeting of the ORF was substantially less effective to restrict virus replication in mosquito-derived cells than target placement in the 3′ NCR^5^. It will be interesting to elucidate whether viral or host factors determine this difference, which might have an influence on a design of safe vaccine candidates. Although the detailed mechanism of miRNA-mediated attenuation of LGTV in the ticks and tick-derived cells was not studied here, we speculate that it occurs in a conventional Dicer dependent/RISC-mediated manner^28^,^29^,^30^,^31^,^32^. Tick-enriched miRNAs (mir-1, -275 or 279) likely guide the RISC onto corresponding complementary target sequence inserted into LGTV genome inducing destabilization/endonucleolytic degradation of viral RNA or its translational repression.

Our data also provides important insights regarding the composition of miRNA targeting cassettes necessary for reliable attenuation of neuropathogenesis of tick-borne flaviviruses. In contrast to non-neurotropic mosquito-borne DEN4^5^ and chimeric TBEV/DEN4^25^ viruses, the insertion of only two miRNA targeting cassettes expressing at least 6 targets for brain tissue-enriched miRNAs in a genome of neurovirulent LGTV is not always sufficient to completely prevent mortality of newborn mice infected by an IC route (Fig. 6F). The residual level of miRNA-targeted virus replication in the CNS (Fig. 6B) possibly provides an opportunity for viruses to escape selective pressure of brain-specific miRNAs by accumulating mutations or deletions in the miRNA target sequence. Nonetheless, virus escape and reversion to a neurovirulent phenotype was reliably diminished by an increase of the number of miRNA-targeting cassettes to sets of 3 that were inserted into distantly located genome regions (Fig. 6F). Moreover, the most effective virus suppression in the brain was achieved when these cassettes included targets for 2 heterologous miRNAs (mir-124 and mir-9) broadly expressed in the CNS. In contrast, the genome targeting for only mir-124 in E(mir) was far less efficient for virus attenuation in the CNS (Fig. 6), which is in an agreement with earlier observations^23^,^33^.

Earlier studies^34^,^35^,^36^ clearly indicated that instability of miRNA-target sequences (such as deletions and/or single nt mismatch between miRNA and its target sequences in viral genome) results in a reversion of the virus to virulent phenotype, which in our case leads to paralysis or death of mice. In this study, neurovirulence of C(mir)/3′(mir) and C(mir)/E(mir)/3′(mir) viruses in newborn mice was evaluated in two independent experiments (Fig. 6F and S6) using a large inoculation dose (10^4^ pfu correspond to 10^6^ IC LD _50_ for the wt LGTV). No morbidity or mortality was observed in any of the 15 animals (10 and 5 mice in 2 experiments, respectively) during the observation period of 21 days. This data indicates that escape mutants do not emerge, otherwise mice would have succumbed to lethal encephalitis as was observed for E(mir)/3′(mir) and C(mir)/E(mir) viruses used in parallel in the study (Fig. 6F and S7). Complemented by the sequencing data (Fig. S6), these results indicate that miRNA targeting regions of C(mir)/3′(mir) and C(mir)/E(mir)/3′(mir) viruses remain stable *in vivo*, which ensures a clearance of these viruses from the CNS as observed on days 13 and 21 pi.

In summary, taken together the results of the present study and our earlier findings on miRNA suppression of mosquito-borne DEN4 in *Aedes* mosquitoes demonstrate that selective miRNA-targeting can be used to achieve simultaneous attenuation of arthropod-borne virus replication in both arthropod vector (mosquitoes or ticks) and vertebrate host. This strategy could potentially be applied for the development of environmentally safe vaccine candidates against many arboviruses from different families. The dual miRNA-based host range restriction of arboviruses could also provide an additional level of biosafety for laboratories or manufacturers of vaccines or diagnostic reagents.

## Materials and Methods

All experimental protocols were approved by the NIH Institutional Biosafety Committee. All animal study protocols were approved by the NIAID/NIH Institutional Animal Care and Use Committee. All animal experiments were performed in compliance with the guidelines of the NIAID/NIH Institutional Animal Care and Use Committee. The NIAID DIR Animal Care and Use Program acknowledges and accepts responsibility for the care and use of animals involved in activities covered by the NIH IRP’s PHS Assurance #A4149-01, last issued 6/11/2011.

### Plasmids and viruses

Infectious full-length cDNA clones of wild type TP-21 (wt) LGTV (GenBank accession number AF825419) and attenuated E5 strains of LGTV under control of Pol II promoter from cytomegalovirus have been described previously^23^. To introduce genetic marker and generate cDNA clone of TP-21 with ablated EcoR*I* restriction site (wt-EcoR* virus), a synonymous G→A substitution was introduced at nt position 8356 of TP-21 genome.

Target sequences for brain- and tick-specific miRNAs as well as their mutated (scrambled) versions are summarized in Supplementary Table S1. To generate a set of 3′(T) viruses carrying a single target in the 3′ NCR, complementary sequence for tick-specific mir-1, mir-184, mir-275, mir-279, or mir-263a miRNA was inserted at nt position 14 of the 3′ NCR of wt LGTV genome. To construct the viruses with multiple targets for homologous (mir-1) or heterologous (mir-1, mir-275, and mir-279) tick-specific miRNAs, 4 sites for target insertions were selected in the 3′ NCR at nt positions 7, 14, 118 and/or 244 of wt TP-21 clone (Fig. 2A). To generate 3′(9/9/9), 3′(124/124/124) and 3′(gf/gf/gf) viruses, three target sequences for mir-1 in 3′(1/1/1) construct were replaced with targets for CNS-specific mir-9 or mir-124, or with sequence corresponding to position 241–260 nts of eGFP coding sequence^37^. To generate 3′(mir) clone carrying sequences complementary to tick- and CNS-specific miRNAs (Fig. 4A), a fused mir-1/mir-9 target sequence was inserted at nt position 10, and two copies of mir-124 target were introduced at nt positions 14 and 244 of the 3′ NCR of LGTV clone E5. Insertion of miRNA target into the dE/NS1R was carried out using polyprotein topology described earlier^24^,^25^. To generate an E(1) clone, a single copy of mir-1 target was fused in-frame with codon-optimized sequence of an E/NS1 stem-anchor region (2171–2488 nts). The resulting fragment was inserted at nt position 2489 of LGTV genome in wt TP-21 clone. Similarly, to generate E(mir) clone, a miRNA targeting cassette containing targets for mir-124/mir-1/mir-124/mir-275/mir-124 was fused with duplicated E/NS1 stem-anchor region and inserted at nt position 2489 of E5 genome. The C(mir) virus that contains miRNA targeting cassette in the dCGR has been described earlier [see C48-124(2)/9/1-E5 virus in ref. 23]. Briefly, the 48 AA N-terminal part of the truncated capsid gene that encodes LGTV 5′ replication promoter was modified by inserting an A residue at nt position 152 of E5 genome. A translational frame (ORF) was restored by inserting a targeting cassette for mir-9, mir-124, mir-1, and mir-124 miRNAs downstream of 5′ promoter region. A codon-optimized copy of full-length C gene was fused with 2A protease gene from foot and mouth disease virus and inserted between miRNA targeting cassette and prM gene of E5 virus cDNA.

Clones encoding combination of miRNA targeting cassettes at dCGR, dE/NS1R, and 3′ NCR of E5 virus (see Fig. 4A) were assembled based on C(mir), E(mir) and 3′(mir) clones using conventional cloning methods^38^. Scrambled control viruses were generated by introducing synonymous mutations in the third nt positions of each AA codon within miRNA target sequences located in the ORF. Sequences of miRNA targets located in the 3′ NCR were substituted with the identical ‘scrambled’ sequences as were the targets located in the ORF. Sequences for all plasmids are available from authors upon request.

### Cells

Vero (African green monkey kidney) cells were maintained in Opti-Pro medium (Invitrogen) supplemented with 2% FBS and 50 μg/ml of gentamicin as previously described^39^. Tick ISE6 cells derived from *Ixodes scapularis*^40^ were maintained at 34 °C in 66% Leibovitz’s L-15 media (Invitrogen) supplemented with 3.3% FBS (Lonza), 6.6% Tryptose phosphate broth solution (MP Biomedicals, Irvine, CA), 0.66% Bovine Cholesterol Lipoprotein Concentrate (MP Biomedicals, Irvine, CA), 50 μg/ml of gentamicin (Invitrogen), 0.296 g/L of L-aspartic acid, 0.333 g/L of L-glutamine, 0.3 g/L of L-proline, 0.323 g/L of L-glutamic acid, 0.296 g/L of α-ketoglutaric acid, 11.9 g/L of D-glucose, and with 1× mineral and vitamin solution as described earlier^41^.

### DNA transfection, virus recovery and titration

Vero cells were seeded onto a 12.5 cm^2^ flask in 5 mL of DMEM supplemented with 10% FBS and 1× Penicillin-Streptomycin-Glutamine (PSG) solution for 24 hrs and were transfected with 5 μg of plasmid DNA using Lipofectamine 2000 reagent (Invitrogen) according to manufacturer’s instructions. Cells were incubated in DMEM at 37 °C and 5% CO_2_ for 5 days, and cell culture aliquots (0.5 mL) were collected daily and stored at −80 °C. Virus titer was determined in Vero cells using an immunostaining plaque-forming assay^39^. For that, each virus was 10-fold serially diluted with Opti-Pro medium in duplicates, and 0.1 mL aliquots were used to infect Vero cells in 24-well plates for 1 hr at 37 °C. One milliliter of Opti-MEM overlay media supplemented with 1% methylcellulose (Invitrogen), 2% FBS, 2 mM L-glutamine, and 50 μg/mL of gentamicin was added to each well, and cells were incubated for 5 days at 37 °C and 5% CO_2_. Overlay media was removed; cells were washed 3 times with PBS and fixed for 20 min with 100% methanol. Foci of infections were visualized using TBEV-specific antibodies in the hyperimmune mouse ascitic fluid and peroxidase-labeled goat anti-mouse IgG (Dako Co., Carpinteria, CA).

### Replication kinetics of LGT viruses in Vero and ISE6 tick cells

Multicycle growth kinetics of LGT viruses in Vero and ISE6 cells were determined as described previously^5^. Briefly, LGTV and its mutants were diluted in Opti-Pro medium supplemented with 2% FBS, 2 mM L-glutamine and used to infect ISE6 or Vero cells at an MOI of 0.01 in duplicate wells (or in single well for data presented in Fig. 1G) of a 6-well plate for 1 h at 34 °C (ISE6) or 37 °C (Vero). Cells were washed three times with Opti-Pro medium and maintained in cell culture media for 5 days at 34 °C (ISE6) or 37 °C (Vero). Aliquots of cell culture medium (0.25 mL) were harvested daily and stored at −80 °C until virus titration. Differences in virus replication kinetics were compared using 2-way ANOVA analysis implemented in Prism 6 software (La Jolla, CA).

### Serial passaging and genetic stability of miRNA targeted viruses in Vero and ISE6 cells

Vero cells in 25 cm^2^ flasks were infected at an MOI of 0.01 with viruses containing single or multiple miRNA targeting cassettes and incubated at 37 °C and 5% CO_2_ for 3 days. Then viruses were harvested, diluted 1:10 with Opti-Pro medium, and 1 mL of virus inoculum was used in a subsequent infection of fresh Vero cells in 25 cm^2^ flasks. The process was repeated 9 times. Cell culture supernatants collected at the end of the 10^th^ passage were processed for RNA extraction using the QIAamp Viral RNA Mini kit (Qiagen). Regions flanking miRNA targeting cassettes were amplified using Titan One Tube RT-PCR kit (Roche, Indianapolis, IN) and sequenced.

ISE6 cells in 25 cm^2^ flasks were infected with miRNA targeted LGTVs at an MOI of 1 and incubated in 5 mL of ISE6 culture medium at 34 °C for 5 days. The virus in cell culture supernatant was collected, and 1 mL of it was used to infect fresh ISE6 cells in 25 cm^2^ flasks. At the end of 2^nd^ ISE6 cells passage RNA was extracted for sequence analysis, and virus titer in the supernatant was determined in Vero cells.

### Competition assay in ISE6 cells

Genetically marked wt-EcoR* virus was mixed at 1:1 ratio (based on pfu) with 3′(1), 3′(184), 3′(275), 3′(279) or 3′(263a) virus. ISE6 cells in 12.5 cm^2^ flasks were infected with viral concoctions at an MOI of 0.1. Cells were washed twice with ISE6 cell culture medium and maintained at 34 °C for 5 days. Viral RNA was extracted from cell supernatants using QIAamp Viral RNA Mini kit (Qiagen), followed by RT-PCR analysis with Com-F2 (5′GTGGAAAGGGACATGAATCA) and Com-R2 (TCCTTAAAGACCCTCTGCTG) primers flanking the cleavage site for EcoR*I* located at nt position 8356. The amplicons of 757 nts were digested with EcoR*I* endonuclease, and DNA products were separated on 1.5% agarose gel followed by staining with ethidium bromide. Images were quantified using TotaLab (version 2.01, TotaLab LTD). Relative fitness for each virus pair was defined as the ratio between signal intensities of DNA fragments derived from wt-EcoR* and miRNA-targeted virus in the sample and their starting ratio in the inoculum used for ISE6 cell infection.

### Synchronous infection of Ixodes ricinus ticks

An *Ixodes ricinus* tick colony was maintained as described previously^42^. Prior to infections, nymphs were transferred to 26 °C for dehydration and desiccation and incubated for 72 hrs at relative humidity less than 80%. For synchronous infection, nymphs were submerged in 1 mL of solution containing ~10^7 ^pfu/mL of virus and incubated at 34 °C for 45 minutes. The ticks were rinsed twice with PBS, dried on Whatman paper, and transferred to glass vials where they were stored inside a desiccator at 26 °C and 97% relative humidity under a 16 hr light/8 hr dark photoperiod. At days 21 and 51 post infection, ticks were frozen and stored at −80 °C. To assess virus infection, ticks were triturated in 0.25 mL of L-15 medium supplemented with 1× SPG (218 mM sucrose, 6 mM L-glutamic acid, 3.8 mM KH_2_PO_4_, 7.2 mM K_2_HPO_4_, pH 7.2), 0.05 mg/mL of Ciprofloxacin, 0.06 mg/mL of Clindamycin and 0.0025 mg/mL of Amphotericin B. Homogenates were centrifuged at 10,000 rpm for 3 min followed by plaque assay in Vero cells. Nymphs were considered infected if at least one virus plaque was detected in undiluted homogenate after 5 days of incubation. Infectivity of miRNA targeted viruses and E5 was compared using one-tailed Fisher’s exact test, followed by p-values adjustment using Bonferroni correction method to account for multiple comparisons.

### Replication kinetics of LGT viruses in the brain of newborn mice

Litters of 10 three-day-old Swiss Webster mice (Taconic, Hudson, NY) were infected intracranially (IC) with 100 pfu of virus in 10 μL of L-15 medium supplemented with 1× SPG as described previously^23^. Brains of 3 mice from each litter were harvested on days 3, 5, and 7 post infection, and a 10% homogenate of each brain was prepared. Virus titers in the brain suspensions were assayed by titration in Vero cells. Statistical comparison of replication kinetics was performed using 2-way ANOVA, followed by p-values adjustment using Tukey’s test implemented in Prism6 software (GraphPad Software, LaJolla, CA).

To evaluate effect of miRNA targeting on neurovirulence of LGTV, 3-day-old Swiss Webster mice were inoculated IC with 0.1 or 1 pfu (for constructs containing single miRNA targeting cassette or scrambled controls), or with 10^3^ or 10^4^ pfu (for constructs containing multiple miRNA targeting cassettes) of recombinant LGT viruses. Mice were returned to cages with their mothers, and monitored for the onset of neurological symptoms (paralysis) daily until 21 dpi, at which point they were humanely euthanized.

To evaluate genetic stability of viruses with multiple miRNA targeting cassettes in the developing CNS, Swiss Webster mice were infected IC with 10^4^ pfu and at 7, 13 and 21 dpi brains from 3 or 5 pups were taken for virus isolation and RT-PCR analysis.

## Additional Information

**How to cite this article**: Tsetsarkin, K. A. *et al*. Concurrent micro-RNA mediated silencing of tick-borne flavivirus replication in tick vector and in the brain of vertebrate host. *Sci. Rep.* **6**, 33088; doi: 10.1038/srep33088 (2016).

## Supplementary Material

Supplementary Information

## References

[b1] Randolph S. E. Tick-borne encephalitis virus, ticks and humans: short-term and long-term dynamics. Curr Opin Infect Dis 21, 462–467 (2008). 18725794 10.1097/QCO.0b013e32830ce74b

[b2] Plotkin S. A. Increasing Complexity of Vaccine Development. J Infect Dis 212 Suppl 1, S12–S16 (2015). 26116724 10.1093/infdis/jiu568

[b3] Seligman S. J. & Gould E. A. Live flavivirus vaccines: reasons for caution. Lancet 363, 2073–2075 (2004). 15207960 10.1016/S0140-6736(04)16459-3

[b4] Pedersen C. E. Jr., Robinson D. M. & Cole F. E. Jr. Isolation of the vaccine strain of Venezuelan equine encephalomyelitis virus from mosquitoes in Louisiana. Am J Epidemiol 95, 490–496 (1972). 4401801 10.1093/oxfordjournals.aje.a121416

[b5] Tsetsarkin K. A. . Dual miRNA targeting restricts host range and attenuates neurovirulence of flaviviruses. PLoS Pathog 11, e1004852 (2015). 25906260 10.1371/journal.ppat.1004852 PMC4408003

[b6] Labuda M. & Nuttall P. A. Tick-borne viruses. Parasitology 129 Suppl, S221–S245 (2004). 15938513 10.1017/s0031182004005220

[b7] Nuttall P. A. & Labuda M. Dynamics of infection in tick vectors and at the tick-host interface. Adv Virus Res 60, 233–272 (2003). 14689696 10.1016/s0065-3527(03)60007-2

[b8] Radda A., Hofmann H. & Kunz C. Viraemia of polecats (Putorius putorius) after infection with tick-borne encephalitis (TE) virus by ticks. Acta Virol 13, 159 (1969). 4389047

[b9] Mandl C. W. . Spontaneous and engineered deletions in the 3′ noncoding region of tick-borne encephalitis virus: construction of highly attenuated mutants of a flavivirus. J Virol 72, 2132–2140 (1998). 9499069 10.1128/jvi.72.3.2132-2140.1998 PMC109508

[b10] Pripuzova N. S. . Safety evaluation of chimeric Langat/Dengue 4 flavivirus, a live vaccine candidate against tick-borne encephalitis. J Med Virol 81, 1777–1785 (2009). 19697399 10.1002/jmv.21587

[b11] Whitehorn J. . Comparative Susceptibility of Aedes albopictus and Aedes aegypti to Dengue Virus Infection After Feeding on Blood of Viremic Humans: Implications for Public Health. J Infect Dis 212, 1182–1190 (2015). 25784733 10.1093/infdis/jiv173 PMC4577038

[b12] Benda R. The common tick Ixodes ricinus L. as a reservoir and vector of tick-borne encephalitis. I. Survival of the virus (strain B3) during the development of the tick under laboratory conditions. Journal of Hygiene, Epidemiology, Microbiology and Immunology 2, 314–330 (1958).

[b13] Rehacek J. Transovarial transmission of tick-borne encephalitis virus by ticks. Acta Virol 6, 220–226 (1962). 14038646

[b14] Singh K. R., Pavri K. & Anderson C. R. Experimental Transovarial Transmission of Kyasanur Forest Disease Virus in Haemaphysalis Spinigera. Nature 199, 513 (1963). 14058628 10.1038/199513a0

[b15] Havlikova S., Lickova M. & Klempa B. Non-viraemic transmission of tick-borne viruses. Acta Virol 57, 123–129 (2013). 23600870 10.4149/av_2013_02_123

[b16] Smith C. E. A virus resembling Russian spring-summer encephalitis virus from an ixodid tick in Malaya. Nature 178, 581–582 (1956). 13369466 10.1038/178581a0

[b17] Price W. H., Thind I. S., Teasdall R. D. & O′Leary W. Vaccination of human volunteers against Russian spring-summer (RSS) virus complex with attenuated Langat E5 virus. Bull World Health Organ 42, 89–94 (1970). 5309521 PMC2427511

[b18] Smorodincev A. A. & Dubov A. V. Tick-borne encephalitis and its vaccino-prophylaxis. (ed. Smorodincev A. A.) 190–211 (Meditsina, Leningrad, 1986).

[b19] Teterina N. L., Maximova O. A., Kenney H., Liu G. & Pletnev A. G. MicroRNA-based control of tick-borne flavivirus neuropathogenesis: Challenges and perspectives. Antiviral Res 127, 57–67 (2016). 26794396 10.1016/j.antiviral.2016.01.003 PMC4760852

[b20] Barrero R. A. . Evolutionary conserved microRNAs are ubiquitously expressed compared to tick-specific miRNAs in the cattle tick Rhipicephalus (Boophilus) microplus. BMC Genomics 12, 328 (2011). 21699734 10.1186/1471-2164-12-328 PMC3141673

[b21] Skalsky R. L., Vanlandingham D. L., Scholle F., Higgs S. & Cullen B. R. Identification of microRNAs expressed in two mosquito vectors, Aedes albopictus and Culex quinquefasciatus. BMC Genomics 11, 119 (2010). 20167119 10.1186/1471-2164-11-119 PMC2834634

[b22] Behura S. K. Insect microRNAs: Structure, function and evolution. Insect Biochem Mol Biol 37, 3–9 (2007). 17175441 10.1016/j.ibmb.2006.10.006

[b23] Tsetsarkin K. A., Liu G., Shen K. & Pletnev A. G. Kissing-loop interaction between 5′ and 3′ ends of tick-borne Langat virus genome ‘bridges the gap’ between mosquito- and tick-borne flaviviruses in mechanisms of viral RNA cyclization: applications for virus attenuation and vaccine development. Nucleic Acids Res (2016). 10.1093/nar/gkw061 PMC4838367 26850640

[b24] Bonaldo M. C. . Construction and characterization of recombinant flaviviruses bearing insertions between E and NS1 genes. Virol J 4, 115 (2007). 17971212 10.1186/1743-422X-4-115 PMC2173888

[b25] Teterina N. L., Liu G., Maximova O. A. & Pletnev A. G. Silencing of neurotropic flavivirus replication in the central nervous system by combining multiple microRNA target insertions in two distinct viral genome regions. Virology 456–457, 247–258 (2014). 10.1016/j.virol.2014.04.001 PMC4075184 24889244

[b26] Pletnev A. G. Infectious cDNA clone of attenuated Langat tick-borne flavivirus (strain E5) and a 3′ deletion mutant constructed from it exhibit decreased neuroinvasiveness in immunodeficient mice. Virology 282, 288–300 (2001). 11289811 10.1006/viro.2001.0846

[b27] Campbell M. S. & Pletnev A. G. Infectious cDNA clones of Langat tick-borne flavivirus that differ from their parent in peripheral neurovirulence. Virology 269, 225–237 (2000). 10725214 10.1006/viro.2000.0220

[b28] Bartel D. P. MicroRNAs: target recognition and regulatory functions. Cell 136, 215–233 (2009). 19167326 10.1016/j.cell.2009.01.002 PMC3794896

[b29] Filipowicz W., Bhattacharyya S. N. & Sonenberg N. Mechanisms of post-transcriptional regulation by microRNAs: are the answers in sight? Nat Rev Genet 9, 102–114 (2008). 18197166 10.1038/nrg2290

[b30] TenOever, B. R. RNA viruses and the host microRNA machinery. Nat Rev Microbiol 11, 169–180 (2013). 10.1038/nrmicro2971 23411862

[b31] Pasquinelli A. E. MicroRNAs and their targets: recognition, regulation and an emerging reciprocal relationship. Nat Rev Genet 13, 271–282 (2012). 22411466 10.1038/nrg3162

[b32] Saetrom P. . Distance constraints between microRNA target sites dictate efficacy and cooperativity. Nucleic Acids Res 35, 2333–2342 (2007). 17389647 10.1093/nar/gkm133 PMC1874663

[b33] Heiss B. L., Maximova O. A., Thach D. C., Speicher J. M. & Pletnev A. G. MicroRNA targeting of neurotropic flavivirus: effective control of virus escape and reversion to neurovirulent phenotype. J Virol 86, 5647–5659 (2012). 22419812 10.1128/JVI.07125-11 PMC3347253

[b34] Heiss B. L., Maximova O. A. & Pletnev A. G. Insertion of microRNA targets into the flavivirus genome alters its highly neurovirulent phenotype. J Virol 85, 1464–1472 (2011). 21123372 10.1128/JVI.02091-10 PMC3028900

[b35] Gitlin L., Stone J. K. & Andino R. Poliovirus escape from RNA interference: short interfering RNA-target recognition and implications for therapeutic approaches. J Virol 79, 1027–1035 (2005). 15613331 10.1128/JVI.79.2.1027-1035.2005 PMC538575

[b36] Yen L. C. . Neurovirulent flavivirus can be attenuated in mice by incorporation of neuron-specific microRNA recognition elements into viral genome. Vaccine 31, 5915–5922 (2013). 22008823 10.1016/j.vaccine.2011.09.102

[b37] Zhang G., Gurtu V. & Kain S. R. An enhanced green fluorescent protein allows sensitive detection of gene transfer in mammalian cells. Biochem Biophys Res Commun 227, 707–711 (1996). 8885998 10.1006/bbrc.1996.1573

[b38] Sambrook J., Fritsch E. & Maniatis T. Molecular Cloning: a Laboratory Manual, (Cold Spring Harbor Laboratory, Cold Spring Harbor, NY, 1989).

[b39] Engel A. R. . The neurovirulence and neuroinvasiveness of chimeric tick-borne encephalitis/dengue virus can be attenuated by introducing defined mutations into the envelope and NS5 protein genes and the 3′ non-coding region of the genome. Virology 405, 243–252 (2010). 20594569 10.1016/j.virol.2010.06.014 PMC2914112

[b40] Munderloh U. G., Liu Y., Wang M., Chen C. & Kurtti T. J. Establishment, maintenance and description of cell lines from the tick Ixodes scapularis. J Parasitol 80, 533–543 (1994). 8064520

[b41] Munderloh U. G. & Kurtti T. J. Formulation of medium for tick cell culture. Exp Appl Acarol 7, 219–229 (1989). 2766897 10.1007/BF01194061

[b42] Bouchard K. R. & Wikel S. K. Care, maintenance, and experimental infestation of ticks in the laboratory setting. In Biology of Disease Vectors. (ed. Marquart W. C.) (Elsevier Academic Press, San Diego, 2005).

