# Supplementary material for: Concurrent micro-RNA mediated silencing of tick-borne flavivirus replication in tick vector and in the brain of vertebrate host
Source: Sci Rep. 2016 Sep 13;6:33088. doi: 10.1038/srep33088 (PMC5020608; doi:10.1038/srep33088)
Supplement: Supplementary Information [file srep33088-s1.doc]

Supplementary Materials for:

**Concurrent micro-RNA mediated silencing of tick-borne flavivirus replication in tick vector and in the brain of vertebrate host**

Konstantin A. Tsetsarkin^1^, Guangping Liu^1^, Heather Kenney^1^, Meghan Hermance^2^, Saravanan Thangamani^3^, Alexander G. Pletnev^1^

Correspondence should be addressed to A.G.P. ([apletnev@niaid.nih.gov](mailto:apletnev@niaid.nih.gov)).

**Supplementary Figure S1. Growth kinetics of LGTV with single copy of miRNA target sequences inserted in the 3’NCR in Vero cells.**

Vero cell monolayers in 12.5 cm^2^ flasks were transfected with 5 µg of indicated DNA constructs. Cell culture medium aliquots with viruses were collected for each indicated time point and titrated in Vero cells in duplicate. Mean virus titers ± SD are shown. The dashed line indicates the limit of virus detection (0.7 log_10_ pfu/mL).

**Supplementary Figure S2. Insertion of multiple copies of target sequences for tick-specific miRNAs into the 3’NCR of LGTV has minimal effect on virus growth in Vero cells.**

Vero cell monolayers in 12.5 cm^2^ flasks were transfected with 5 µg of indicated DNA constructs. Virus aliquots in cell culture medium were collected for each indicated time point and titrated in Vero cells in duplicate.

**Supplementary Figure S3. Deletion mutations found in the 3’NCR after 2^nd^ passage of miRNA targeted viruses in ISE6 cells.**

(**A**) top: schematic representation of experiment. To initiate virus replication in tick cells, ISE6 cells were infected at an MOI of 1. Viral RNA was extracted from cell culture supernatants collected after 2^nd^ passage in ISE6 cells. RNA was used for RT-PCR analysis with primers flanking sites of miRNA targets insertion. Bottom: RT- PCR products were separated using 1% agarose gel and stained with ethidium bromide. (L) stands for the longer RT- PCR product. (S) stands for the shorter RT- PCR product. The selected bands of 1 kb DNA ladder (GeneRuler, Thermo Scientific) are indicated. (**B**) RT- PCR products were extracted from agarose gel and sequenced. Location and size of the deletions (strikethrough lines) for each of RT- PCR products are indicted.

**Supplementary Figure S4. Growth of 3’(1/1/1), 3’(9/9/9), 3’(124/124/124) and 3’(gf/gf/gf) in ISE6 cells and brains of newborn mice.**

(**A**) Schematic representation of recombinant LGTV genomes used in this study. Top: diagram indicating sites for miRNA target insertions in the predicted secondary structure of the 3’NCR of LGTV. Bottom: recovered viruses with specific composition of the miRNA target(s) inserted into the 3’NCR. (**B**) Growth kinetics of wt-EcoR* and miRNA targeted LGT viruses in ISE6 cells infected at an MOI of 0.01. (C) Mean virus titer (n=3 per time point) in the brain of newborn Swiss mice infected IC with 100 pfu. Brains were collected on 3, 5, and 7 dpi.

**
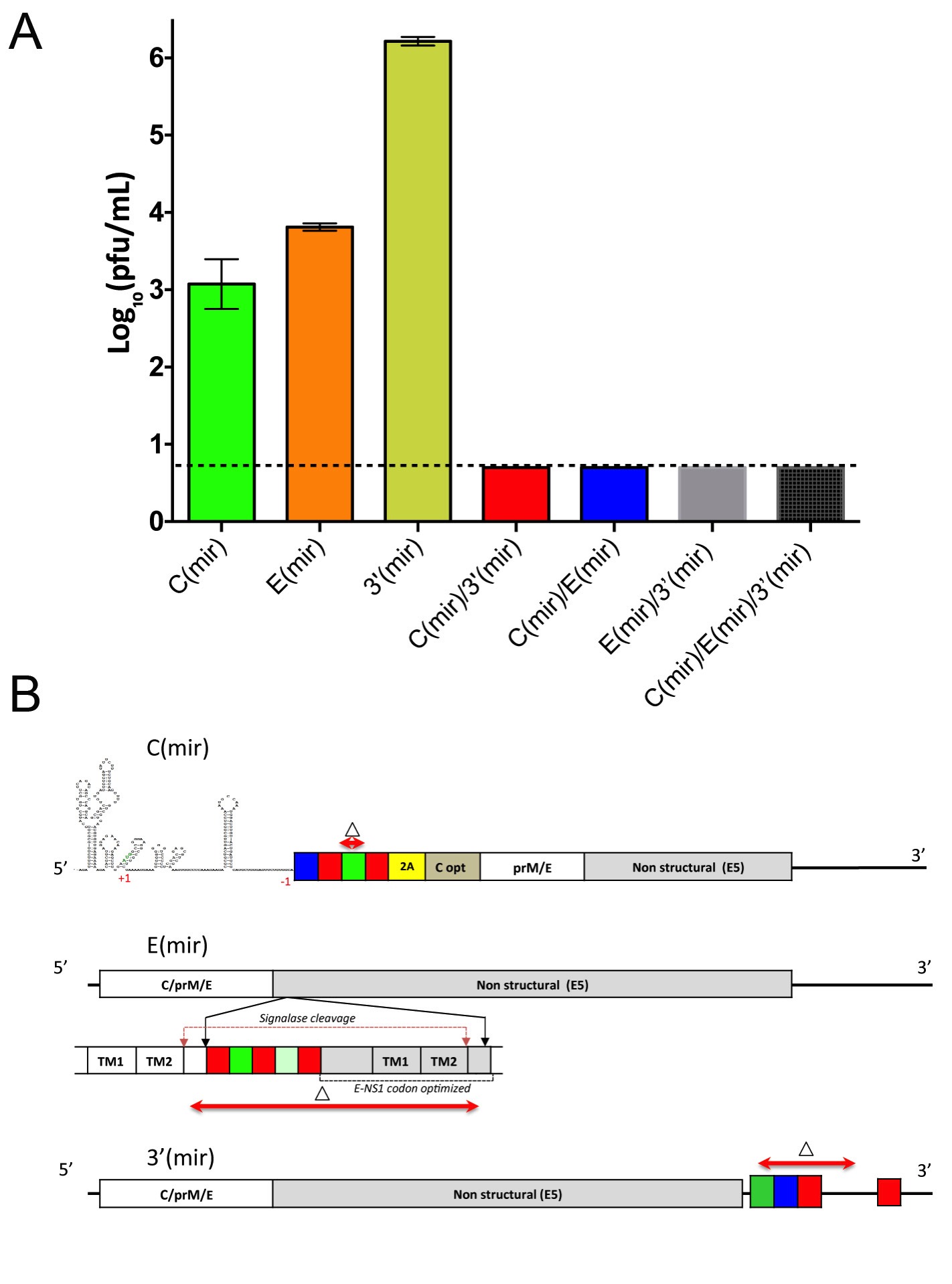
**

**Supplementary Figure S5. Targeting of the distant LGTV genome regions for tick-specific miRNAs is more effective to control virus replication in tick derived cells than targeting of only single site.**

(**A**) The titer (± standard deviation) of miRNA targeted viruses after 2^nd^ passage in ISE6 cells. Virus titer was determined in two replicates in Vero cells. To initiate replication, ISE6 cells were infected at an MOI of 1.

(**B**) Schematic location of the deletion mutants (red double arrows) in genome of C(mir), E(mir), and 3’(mir) viruses which were recovered after 2^nd^ passage in ISE6 cells. Viral RNA was extracted from cell culture and was used for RT-PCR analysis with primers flanking sites of miRNA targets insertion, followed by cDNA sequencing.

**Supplementary Figure S6. Stability of C(mir)/3’(mir) and C(mir)/E(mir)/3’(mir) viruses in the CNS of mice.**

Three-day-old Swiss Webster mice (Taconic, Hudson, NY) were infected IC with 10^4^ pfu of C(mir)/3’(mir) [**A**] or C(mir)/E(mir)/3’(mir) viruses [**B**]. At 7, 13 and 21 dpi brains from 3 or 5 pups were taken for virus isolation and RT-PCR analysis with primers flanking sites of miRNA targets insertion, followed by cDNA sequencing. Virus titers in the 10% brain suspensions were assayed by titration in Vero cells.

‘Stable’ - mutations or deletions were not detected at any of the miRNA targeting regions.

‘N/A’ - not analyzed due to undetectable virus load in the CNS

**Supplementary Figure S7. Stability of C(mir)/E (mir) and E(mir)/3’(mir) viruses in the CNS of mice.**

Three-day-old Swiss Webster mice (Taconic, Hudson, NY) were infected IC with 10^4^ pfu of C(mir)/E (mir) [**A**], or E(mir)/3’(mir) viruses [**B**]. At 7, 13 and 21 dpi brains from 3 or 5 pups were taken for virus isolation and RT-PCR analysis with primers flanking sites of miRNA targets insertion, followed by cDNA sequencing.

At the top is a table showing viral load and summary of sequencing results for viruses isolated from individual mouse brains.

‘Stable’ – point mutation(s) or deletions were not detected at any of the miRNA targeting regions.

‘Not stable’ - point mutation(s) and/or deletions were detected in at least one of the miRNA targeting regions. Numbers in parentheses provide samples reference for the figure at the bottom.

‘N/A’ - not analyzed due to undetectable virus load in the brain.

* - denotes the samples obtained from the brain of paralyzed mouse.

At the bottom is a schematic location of the point mutations (**#**) and/or deletions (red double arrows) in the ‘not stable’ viral genomes.

**Table S1. Summary of miRNA target sequences used in the study**.

| miRNA Target | Sequences 5’🡪3’ ^a^ | Color code used in the paper | Specificity | Length (nt.) |
| --- | --- | --- | --- | --- |
| mir-1 | CTCCATACTTCTTTACATTCCA | Green / solid | tick | 22 |
| mir-1* | tTgCAcACgagccTtCAcagtA | Green / stripes | tick | 22 |
| mir-184 | GCCCTTATCAGTTCTCCGTCCA | Green / solid | tick | 22 |
| mir-275 | CGCGCGCTACTTCAGGTACCTGA | Green / solid | tick | 23 |
| mir-275* | aGaGCatTgtTgCAaGTtCCaGA | Green / stripes | none | 23 |
| mir-279 | TGGATGAGTGTGGATCTAGTCA | Green / solid | tick | 22 |
| mir-263a | CCGTGAATTCTTCCAGTGCCATT | Green / solid | tick | 23 |
| mir-124 | GGCATTCACCGCGTGCCTTA | Red / solid | brain | 20 |
| mir-124* | GcCAcaGtCCtaGgGCacTg | Red / stripes | none | 20 |
| mir-9 | TCATACAGCTAGATAACCAAAGA | Blue / solid | brain | 23 |
| mir-9* | TgATcCAatTgGAcAAtCAgAGg | Blue / stripes | none | 23 |
| gfp | CAGCACGACTTCTTCAAGTC | N/A | none | 20 |

^a^ Lowercase letters indicate synonymous mutations that were incorporated to alter nucleotide sequence of target miRNA region in control viruses.

N/A – not applicable (color coding was not utilized for schematic representation of 3’(gf/gf/gf) vuirs)
